# A multi-network clustering method for detecting protein complexes from multiple heterogeneous networks

**DOI:** 10.1186/s12859-017-1877-4

**Published:** 2017-12-01

**Authors:** Le Ou-Yang, Hong Yan, Xiao-Fei Zhang

**Affiliations:** 1 College of Information Engineering & Shenzhen Key Laboratory of Media Security, Shenzhen University, Nanhai Ave 3688, Shenzhen, 518060 China; 2 Department of Electronic and Engineering, City University of Hong Kong, Tat Chee Avenue, Hong Kong, China; 3 School of Mathematics and Statistics & Hubei Key Laboratory of Mathematical Sciences, Central China Normal University, Wuhan, 430079 China

**Keywords:** Protein-protein interaction, Domain-domain interaction, Protein complex, Multi-network clustering

## Abstract

**Background:**

The accurate identification of protein complexes is important for the understanding of cellular organization. Up to now, computational methods for protein complex detection are mostly focus on mining clusters from protein-protein interaction (PPI) networks. However, PPI data collected by high-throughput experimental techniques are known to be quite noisy. It is hard to achieve reliable prediction results by simply applying computational methods on PPI data. Behind protein interactions, there are protein domains that interact with each other. Therefore, based on domain-protein associations, the joint analysis of PPIs and domain-domain interactions (DDI) has the potential to obtain better performance in protein complex detection. As traditional computational methods are designed to detect protein complexes from a single PPI network, it is necessary to design a new algorithm that could effectively utilize the information inherent in multiple heterogeneous networks.

**Results:**

In this paper, we introduce a novel multi-network clustering algorithm to detect protein complexes from multiple heterogeneous networks. Unlike existing protein complex identification algorithms that focus on the analysis of a single PPI network, our model can jointly exploit the information inherent in PPI and DDI data to achieve more reliable prediction results. Extensive experiment results on real-world data sets demonstrate that our method can predict protein complexes more accurately than other state-of-the-art protein complex identification algorithms.

**Conclusions:**

In this work, we demonstrate that the joint analysis of PPI network and DDI network can help to improve the accuracy of protein complex detection.

## Background

Proteins seldom act alone, they tend to interact with each other and form protein complexes to perform their functions [1, 2]. The identification of protein complexes is essential for the understanding of cellular organization and function [3–5]. Although some biological experiment methods, such as Tandem Affinity Purification (TAP) with mass spectrometry [6, 7] and Protein-fragment Complementation Assay (PCA) [8], have been developed to detect protein complexes, these methods have some inevitable limitations such as low-throughput outcome [3, 9]. Due to these limitations, the number of known protein complexes is still limited. Therefore, computational detection of protein complexes, which can be acted as useful complements to the experiment methods, is quite necessary [10–15].

In recent years, high-throughput experimental techniques have been developed to identify protein-protein interactions (PPI). The accumulation of PPI data facilitates the development of computational approaches for protein complex identification [9, 16]. A PPI network is usually modelled as an undirected graph, where nodes represent proteins and edges represent protein-protein interactions. Since proteins within same protein complexes tend to interact with each other, dense regions in PPI networks may be potential protein complexes. Based on this assumption, various graph clustering algorithms have been developed to identify protein complexes from PPI networks, such as MCODE [17], CFinder [18], MCL [19], RNSC [20], COACH [21], ClusterONE [22]. However, PPI data collected by high-throughput methodologies are known to be quite noisy. It is hard to achieve reliable prediction results by simply apply graph clustering algorithms on PPI data.

Protein domains are structural (or functional) subunits that make up proteins [23]. The interaction between two proteins typically involves the physical interaction between specific protein domains [24]. Understanding protein interactions at the domain level can give us a global view of protein functions and the PPI network [25–27]. In recent years, several databases, such as the Protein families (Pfam) [28], have compiled comprehensive information about protein domains. The availability of protein domain information makes it possible for us to utilize domain-protein associations and domain-domain interactions (DDI) to evaluate the propensities of proteins pairs to interact. Therefore, the joint analysis of PPIs, domain-protein associations and DDIs has the potential to improve the accuracy of protein complex detection [29]. However, existing protein complex identification methods are primary designed for detecting protein complexes from a single PPI network. Although some multi-view graph clustering algorithms have been developed for clustering multiple networks, most of the existing methods are based on the assumption that information collected from different data sources consist of the same set of instances, which means different networks denote different representations of a same set of instances [30–33]. Given that most proteins are multi-domain proteins, we need to design an algorithm that can generalized multi-view graph clustering to allow many-to-many relationships between the nodes in different networks, and jointly analyze multiple networks consist of different sets of instances and have different sizes [34, 35].

**Fig. 1:**
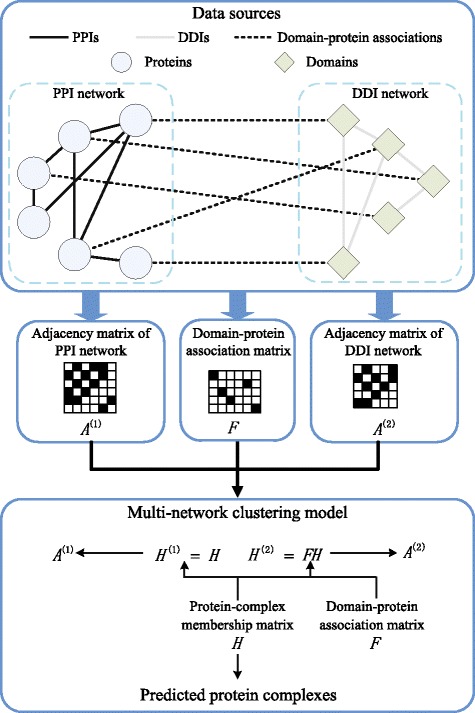
Schematic overview of the algorithm. The flowchart of our multi-network clustering procedure for detecting protein complexes

To address the above challenges, in this study, we introduce a novel multi-network clustering (MNC) model to exploit the shared clustering structure in PPI and DDI networks to improve the accuracy of protein complex detection. The overall framework of our algorithm is shown in Fig. 1. Unlike previous multi-view clustering algorithms that assume all views consist of the same set of instances, our method is a flexible approach that allows different networks to have different instances and different sizes. In particular, we consider the case when the networks are collected from different but related fields (i.e., PPI network and DDI network), and the cross-field instance relationship is many-to-many (i.e., a protein may contain multiple domains). Given a PPI network and a DDI network, we first introduce a generative model to describe the generation processes of these two networks. Secondly, based on the domain-protein associations, the generation processes of PPI and DDI networks are assumed to be dominated by a shared clustering structure, which describes the degree of proteins belonging to complexes. Finally, the protein complex detection problem is transformed into a parameter estimation problem. We have conducted comprehensive experiments to evaluate the performance of various protein complex detection algorithms. The experiment results demonstrate that by incorporating domain interactions and domain-protein associations, our multi-network clustering algorithm could generate more reliable prediction results than other state-of-the-art protein complex detection algorithms.

## Methods

In this section, we describe our multi-network clustering (MNC) model as shown in Fig. 1 in details.

### Model formulation

Given a PPI network *G*_1_ with *N*_1_ proteins and a DDI network *G*_2_ with *N*_2_ domains, two nonnegative score matrices $A^{(1)}\in \mathbb {R}_{+}^{N_{1} \times N_{1}}$ and $A^{(2)}\in \mathbb {R}_{+}^{N_{2} \times N_{2}}$ are used to represent the affinity/adjacency matrix of *G*_1_ and *G*_2_ respectively. Note that *G*_1_ represents a PPI network and *G*_2_ represents a DDI network, the two adjacency matrices *A* ^(1)^ and *A* ^(2)^ may have different dimensions, i.e. *N*_1_≠*N*_2_, and the relationships between nodes in *G*_1_ and nodes in *G*_2_ may be many-to-many. The domain-protein associations can be described by a *N*_2_×*N*_1_ matrix *F*, where *F*_*xi*_=1 if protein *i* in *G*_1_ contains domain *x* in *G*_2_, and *F*_*xi*_=0 otherwise. Our goal is to jointly exploit the clustering structures in PPI network *G*_1_ and DDI network *G*_2_, and infer $H_{ik}^{(m)}$ which describes the weight of node *i* in the predicted *k*-th cluster of *m*-th network from each network *A* ^(*m*)^ (a higher value of $H_{ik}^{(m)}$ represents that node *i* is more likely to belong to cluster *k*, and vice versa).

Suppose there are *K*_*m*_ clusters in network *G*_*m*_. According to the definition of *A* ^(*m*)^ and *H* ^(*m*)^, $W_{ij}^{(m)} = 1 - \exp \left (- \sum _{k=1}^{K_{m}} H_{ik}^{(m)}H_{jk}^{(m)}\right)$ represents the underlying co-cluster affinity between nodes *i* and *j* and each element $A_{ij}^{(m)}$ of *A* ^(*m*)^ represents the observed interaction between nodes *i* and *j*, where $A_{ij}^{(m)} = 1$ if there is an edge between nodes *i* and *j* and $A_{ij}^{(m)} = 0$ otherwise. Thus, based on the assumption that if two nodes are connected in a network, they are more likely to belong to same clusters, we could infer the underlying clusters *H* ^(*m*)^ based on the observed data *A* ^(*m*)^. In particular, given *H* ^(*m*)^, we can write down the following probability of generating a particular network *A* ^(*m*)^:

1 $$ { \begin{aligned} P\left(A^{(m)}|H^{(m)}\right) &= \prod\limits_{ij} {W_{ij}^{(m)}}^{A_{ij}^{(m)}} \left(1 - W_{ij}^{(m)}\right)^{\left(1 - A_{ij}^{(m)}\right)}\\ &= \prod\limits_{ij} \left[1 - \exp\left(- \sum\limits_{k=1}^{K_{m}} H_{ik}^{(m)}H_{jk}^{(m)}\right)\right]^{A_{ij}^{(m)}}\\ &{\exp\left(- \sum\limits_{k=1}^{K_{m}} H_{ik}^{(m)}H_{jk}^{(m)}\right)}^{\left(1 - A_{ij}^{(m)}\right)}. \end{aligned}}  $$

In this study, we focus on exploiting the underlying common clustering patterns of different heterogeneous networks. As an interaction between two proteins typically involves physically interacting between specific protein domains, there may be some matching relationships between the clusters in PPI networks and the clusters DDI networks. Therefore, in this study, based on the domain-protein association matrix *F*, *H* ^(2)^ is defined as *F* *H* ^(1)^, where $H_{xk}^{(2)} = \sum _{i=1}^{N_{1}} F_{xi} H_{ik}^{(1)}$. With this definition, the predicted memberships of a domain are consistent with the predicted memberships of the proteins that contain this domain. To describe the relationship between *H* ^(1)^ and *H* ^(2)^, we introduce a nonnegative matrix $H \in \mathbb {R}_{+}^{N_{1}\times K}$ and set *H* ^(1)^=*H* and *H* ^(2)^=*F* *H* ^(1)^=*F* *H*.

Similar to [36], nonnegative priors for *H* are chosen to make sure that all elements of *H* are nonnegative. Specifically, independent Half-Normal priors with zero mean and variance *λ*=[*λ*_*k*_] are assigned on each column of *H*:

2 $$ \begin{aligned} P(H_{ik}|\lambda_{k}) &= \mathcal{HN}(H_{ik}|\lambda_{k}), \quad \text{for} \quad i = 1, \ldots, N_{1},\\ k &= 1, \ldots, K. \end{aligned}  $$

where for *u*≥0, $\mathcal {HN}(u|\sigma) = \left (\frac {2}{\pi \sigma }\right)^{1/2} \exp \left (-\frac {u^{2}}{2\sigma }\right)$, and $\mathcal {HN}(u|\sigma) = 0$ when *u*<0. We can find from Eq. (2) that all elements of the *k*-th column of *H* are associated with a same variance parameter *λ*_*k*_ which controls the relevance of the corresponding cluster in accounting for the observed interactions. When the value of *λ*_*k*_ is small, all elements of the *k*-th column of *H* are close to zero, which means the *k*-th column of *H* is not relevant and can be removed from the factorization. Through this filter, we could obtain a more parsimonious model which indicates the optimal number of clusters.

Finally, an inverse-Gamma prior, which is a conjugate prior for the Half-Normal distribution, is assigned to each relevance weight *λ*_*k*_:

3 $$ P(\lambda_{k}; a, b) = \frac{b^{a}}{\Gamma(a)}\lambda_{k}^{-(a+1)} \exp\left(-\frac{b}{\lambda_{k}}\right).  $$

where *a*>0 and *b*>0 are the shape and scale parameters respectively. In this study, the values of *a* and *b* are fixed for all *λ*_*k*_. Based on the independence assumption of *H* and *λ*, we consider the following generation process of networks *G*_1_ and *G*_2_:

4 $$ \begin{aligned} P\left(A^{(1)}, A^{(2)}, H, \lambda|F\right) &= P\left(A^{(1)}|H\right)P(A^{(2)}|F,H)\\&\quad \,P(H|\lambda)P(\lambda). \end{aligned}  $$

where *P*(*A* ^(1)^|*H*) and *P*(*A* ^(2)^|*F*,*H*) are defined in Eq. (1) and

5 $$ P(H|\lambda) = \prod\limits_{i,k} \left(\frac{2}{\pi \lambda_{k}}\right)^{1/2} \exp\left(-\frac{H_{ik}^{2}}{2\lambda_{k}}\right),  $$

6 $$ \begin{array}{ll} P(\lambda) = \prod\limits_{k = 1}^{K} P(\lambda_{k}; a, b) = \prod\limits_{k=1}^{K} \frac{b^{a}}{\Gamma(a)}\lambda_{k}^{-(a+1)} \exp\left(-\frac{b}{\lambda_{k}}\right). \end{array}  $$

With the observed networks *A* ^(1)^ and *A* ^(2)^, the values of the model parameters *H* and *λ* can be estimated by maximizing the joint probability (4). By substituting Eqs. (1), (5) and (6) into Eq. (4), and taking the negative logarithm and dropping constants, the objective function of our proposed multi-network clustering (MNC) model is formulated as follows:

7 $$ \begin{array}{ll} \min\limits_{H,\lambda} - \log P\left(A^{(1)}, A^{(2)}, H, \lambda|F\right) \\ = - \log P\left(A^{(1)}|H\right) - \log P\left(A^{(2)}|F,H\right) - \log P(H|\lambda)\\ \quad- \log P(\lambda) \\ = - \sum_{i,j=1}^{N_{1}} A_{ij}^{(1)} \log\left[1 - \exp\left(- \sum_{k=1}^{K} H_{ik}H_{jk}\right)\right] \\ \quad + \sum_{i,j=1}^{N_{1}} \left(1 - A_{ij}^{(1)}\right) \sum_{k=1}^{K} H_{ik}H_{jk}\\ \quad - \sum_{x,y=1}^{N_{2}} A_{xy}^{(2)} \log\left[1 - \exp\left(- F H H^{T} F^{T}\right)_{xy}\right] \\ \quad + \sum_{x,y=1}^{N_{2}} \left(1 - A_{xy}^{(2)}\right) \left(F H H^{T} F^{T}\right)_{xy} \\ \quad + \sum_{i=1}^{N_{1}}\sum_{k=1}^{K} \frac{1}{2\lambda_{k}} \left(H_{ik}\right)^{2} + \frac{N_{1}}{2}\sum_{k=1}^{K} \log{\lambda_{k}}\\ \quad+ \sum_{k=1}^{K} \frac{b}{\lambda_{k}} + (a+1)\sum_{k=1}^{K} \log{\lambda_{k}}, \\ s.t.\quad\quad\quad H\geq 0, \end{array}  $$

where *H*≥0 means each element *H*_*ik*_≥0.

### Parameter estimation

An alternating optimization scheme is adopted to solve the objective function in Eq. (7). Specifically, we optimize the objective function in Eq. (7) with respect to one variable while fixing others. According to the multiplicative update rule [37, 38], we can obtain the following two updating rules for *H*_*ik*_ and *λ*_*k*_:

8 $$ \lambda_{k} \leftarrow \frac{2b + \sum_{i=1}^{N_{1}} H_{ik}^{2}}{N_{1} + 2a + 2}.  $$

and

9 $$ {\begin{aligned} H_{ik} \leftarrow \frac{H_{ik}}{2} + \frac{H_{ik}}{2} \frac{\sum\limits_{j=1}^{N_{1}} \frac{A_{ij}^{(1)}H_{jk}}{1 - exp(-HH^{T})_{ij}} + \sum\limits_{x,y=1}^{N_{2}} \frac{A_{xy}^{(2)} F_{xi}\sum\limits_{j=1}^{N_{1}} H_{jk}F_{yj}}{1-exp(-FHH^{T}F^{T})_{xy}}}{\sum\limits_{j=1}^{N_{1}} H_{jk} + \sum\limits_{x,y=1}^{N_{2}} F_{xi} \sum\limits_{j=1}^{N_{1}} H_{jk}F_{yj} + \frac{1}{2\lambda_{k}}H_{ik}}, \end{aligned}}  $$

Once *H* is initialized, we update *λ* and *H* according to Eqs. (8) and (9) alternately until a stopping criterion has been satisfied. Note that the objective function is not jointly convex with respect to all variables. Thus, the final estimators of *H* and *λ* depend on the initial value of *H*. Proper initialization is therefore needed to achieve satisfactory performance. In this study, a heuristic method is utilized to initialize *H*. That is, we utilize the clustering result of a chosen algorithm (i.e., MCL) on PPI network *G*_1_ to generate the initial value of *H*. We first utilize the chosen algorithm to detect $\hat {K}$ clusters from network *G*_1_, which involve $\hat {N}$ nodes, then we set each of the remaining $N_{1} - \hat {N}$ unclustered nodes to be a singleton cluster. Finally, this initialization clustering result is converted into an $N_{1} \times (\hat {K} + N_{1} - \hat {N})$ binary indicator matrix *H* ^*initial*^, where:

10 $$ H_{ik}^{initial} = \left \{ \begin{array}{cc} 1, & \text{if node} \,i \, \text{is assigned to cluster}\, k,\\ 0, & \text{otherwise}. \end{array}\right.  $$

Similar to [39], a small positive perturbation is added to all entries of *H* ^*initial*^ and the resulting perturbed matrix is used to feed our optimization algorithm. In practice, we stop the iteration process when the relative change of the objective function (7) is less than 10^−3^.

### Protein complex detection

After obtaining the final estimator $\hat {H}$, as all elements of $\hat {H}$ are nonnegative real values, we need to transform $\hat {H}$ into a final protein-complex assignment matrix *H* ^⋆^. Similar to [40, 41], we transform $\hat {H}$ into *H* ^⋆^ by taking a threshold *τ*. In particular, we assign protein *i* to complex *k* if $\hat {H}_{ik}$ exceeds *τ*. That is, we set $H_{ik}^{\star } = 1$ if *H*_*ik*_≥*τ* and set $H_{ik}^{\star } = 0$ if *H*_*ik*_<*τ*. Here, $H_{ik}^{\star } = 1$ indicates that protein *i* is assigned to predicted complex *k*. In practice, we have found that *τ*=0.3 always leads to reasonable results [41, 42], so we set *τ*=0.3 in this study. The procedure of our multi-network clustering (MNC) algorithm is summarized in Algorithm 1.



## Results

### Experimental Datasets

In this study, we employ two heterogeneous networks for yeast, i.e., a PPI network and a DDI network, to evaluate the performance of various protein complex detection algorithms. The PPI data is downloaded from the DIP database [43], which involves with 17,201 protein interactions among 4930 proteins. The DDI data and domain-protein association data are downloaded from the following three databases, namely 3DID [44], iPfam [45] and DOMINE [23], which involves with 4781 domain interactions among 1256 domains and 2613 domain-protein associations between 1256 domains and 1948 proteins. We employ 3 benchmark complex sets, namely CYC2008 [46], MIPS [47] and SGD [48], as gold-standards. For each benchmark complex set, proteins that are not involved in the PPI data are filtered out. Furthermore, as suggested by Nepusz et al. [22], only complexes with at least three proteins are considered. As a consequence, CYC2008 contains 226 complexes covering 1190 proteins, MIPS contains 200 complexes covering 1059 proteins and SGD contains 230 complexes covering 1103 proteins. We also utilize the Gene Ontology (GO) functional annotations of yeast to evaluate the functional homogeneity of our predicted novel complexes. The GO file contains three types of annotations, i.e., molecular function, biological process and cellular component [49].

### Evaluation metrics

In this study, we use two independent evaluation metrics to assess the performance of various protein complex identification algorithms. The first evaluation metric is the geometric accuracy (Acc) as introduced by Xie et al. [50], which is the geometric mean of sensitivity (Sn) and positive predictive value (PPV). Given a known complex *b*_*i*_ and a predicted complex *q*_*j*_, let *T*_*i*,*j*_ denote the number of proteins shared by *b*_*i*_ and *q*_*j*_. Sn, PPV and Acc are defined as follows:

11 $$\begin{array}{@{}rcl@{}} && Sn = \frac{\sum_{i} \max_{j} T_{i, j}}{\sum_{i}|b_{i}|}, PPV = \frac{\sum_{j} \max_{i} T_{i, j}}{\sum_{j} |\cup_{i}(b_{i} \cap q_{j})|}, \\ && Acc = \sqrt{Sn \times PPV}  \end{array} $$

where |·| counts the elements within a given set. The second evaluation metric is the fraction of matched complexes (FRAC) [22], which calculates the percentage of benchmark complexes that are identified. Given *b*_*i*_ and *q*_*j*_, their overlapping score (OS) is defined as follows:

12 $$ OS(b_{i}, q_{j}) = \frac{|b_{i}\cap q_{j}|^{2}}{|b_{i}||q_{j}|}.  $$

We consider *b*_*i*_ and *q*_*j*_ to be matching if *O* *S*(*b*_*i*_,*q*_*j*_)≥*ω*. Similar to other researches [41, 42], we set the value of *ω* to be 0.25. The definition of FRAC is shown in Eq. (13), where *B* is the set of benchmark complexes and *Q* is the set of predicted complexes.

13 $$ FRAC = \frac{|\{b_{i}|b_{i}\in B \wedge \exists q_{j}\in Q, q_{j}\,\,matches\,\,b_{i}\}|}{|B|}.  $$

Besides Acc and FRAC, other quality metrics, such as Precision, Recall and F-measure, are also widely used to evaluate the performance of a clustering algorithm. Let *TP* (true positive) denote the number of predicted complexes that are matched by the benchmark complexes, and *FN* (false negative) denote the number of benchmark complexes that are not matched by any of the predicted complexes, and *FP* (false positive) denote the number of predicted complexes minus *TP*. Precision, Recall and F-measure are defined as follows:

14 $$\begin{array}{@{}rcl@{}} && Recall = \frac{TP}{TP + FN}, Precision = \frac{TP}{TP + FP}, \\ && F-measure = \frac{2\times Precision \times Recall}{Precision + Recall}. \end{array} $$

Note that the reference data sets are far from complete. In particular, the PPI data used in our study covers 4930 proteins, whereas the three benchmark complex sets, namely, CYC2008, MIPS and SGD, only cover 1190, 1059 and 1103 proteins respectively. Thus, predicted protein complexes that do not match with any known complexes are not necessarily undesired results. On the contrary, they may be potential protein complexes [22]. As optimizing Precision and F-measure will somehow prevent us from detecting novel complexes, we do not use these evaluation metrics in this study.

As the reference data sets are incomplete, following the method of Nepusz et al. [22], we also evaluate the functional homogeneity of our predicted complexes. We use the hypergeometric distribution to calculate the *P*-value of biological relevance for a predicted complex and a given functional term. Suppose the background set covers *N* proteins. Given a predicted complex which includes *C* proteins and a functional group which contains *S* proteins. Suppose that *z* proteins in the functional group are included in the predicted complex, then *P*-value focus on calculating the probability of observing *z* or more proteins in the functional group that are included the predicted complex by chance:

15 $$ P-value = 1 - \sum\limits_{l=1}^{z-1} \frac{\left(\begin{matrix} S\\ l \end{matrix}\right)\left(\begin{matrix} N - S\\ C - l \end{matrix}\right)}{\left(\begin{matrix} N\\ C \end{matrix}\right)}  $$

### Parameter settings

Our model has two parameters *a* and *b* that need to be predefined. The effect of parameter *a* is implied in the updating rule (8). As shown in Eq. (8), the influence of *a* can be moderated by the number of proteins *N*_1_. Therefore, following [42], we fix the value of *a* to be 2 and vary the value of *b* to evaluate the effect of this parameter. Although the reference data sets are far from complete, we can still use some of the known complexes to do parameter selection. In this study, the MIPS benchmark complex set is used to test the effect of parameters. Since most of the existing protein complex identification algorithms need to do parameter selection, we also utilize MIPS benchmark complex set to select the optimal parameters for these algorithms.

**Fig. 2:**
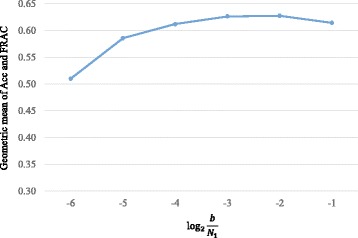
The effect of *b*. Performance of MNC on protein complex identification with different values of *b* measured by geometric mean of Acc and FRAC with respect to MIPS benchmark complex set. The *x*-axis denotes the value of $\log \frac {b}{N_{1}}$ and the *y*-axis denotes the geometric mean of Acc and FRAC

In particular, we vary the value of *b* (*b*∈{*N*_1_×2^−6^,*N*_1_×2^−5^,…,*N*_1_×2^−1^}), and assess how well the predicted complexes match with MIPS benchmark complex set. We use the geometric mean of Acc and FRAC the measure the performance of our method. We can find from Fig. 2 that as the value of *b* increases, the geometric mean scores increase initially and decrease after reaching the maximum. Overall, with respect to MIPS benchmark complex set, *b*=*N*_1_×2^−2^ would be the optimal setting for *b*. In the following experiments, we keep *a*=2 and *b*=*N*_1_×2^−2^ as the default values of our method.

### Comparisons with state-of-the-art protein complex detection algorithms

To demonstrate the effectiveness of our model in detecting protein complexes, we compare our MNC with seven existing state-of-the-art protein complex identification algorithms, including CFinder [18], ClusterONE [22], CMC [51], MCL [19], RNSC [20], RRW [52] and SPICi [53]. As traditional protein complex identification algorithms are designed for mining clusters in a single PPI network, we apply the above algorithms on PPI network and apply our method on PPI and DDI networks. For a fair comparison, following the strategy used in [22, 33], for each compared algorithm, optimal parameters with respect to the MIPS benchmark complex set are set to generate its best results. Note that in this study, we initialize the model parameter *H* of MNC based on the clustering result of MCL on PPI network. Moreover, for all the compared algorithm, the predicted complexes with less than three proteins are discarded.

**Fig. 3:**
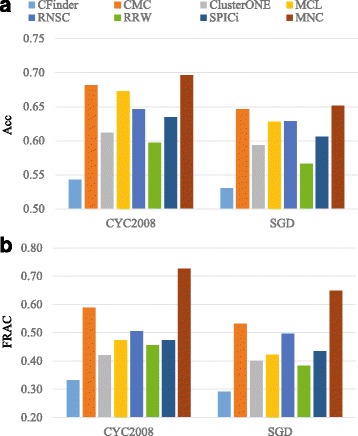
Comparison with existing protein complex identification algorithms. Performance of existing algorithms and our method in terms of (**a**) Acc and (**b**) FRAC, with respect to CYC2008 and SGD

The performances of different protein complex identification algorithms are shown in Fig. 3. We can find that our MNC achieves better performance than other seven compared algorithms in terms of all evaluation metrics, with respect to CYC2008 and SGD. For example, with respect to CYC2008, MNC achieves Acc 0.697 and FRAC 0.726, which is 2.2% and 23% higher than the second best Acc and FRAC achieved by CMC. As shown in Fig. 3, the obvious performance difference between MNC and MCL (which is used to generate the initial value for the model parameter of MNC) indicates that the performance superiority of MNC is owing to the nature of our proposed model but not to the initialization conditions. In Table 1, we present the results of our model with random initial conditions (initialize matrix *H* randomly with *K*=1500). As shown in Table 1, there is no significant performance difference between MNC and MNC _*rand*_, which means that the performance of MNC does not heavily rely on the initialization of *H*. However, when using the clustering results of MCL to initialize *H*, the complexes predicted by MNC can cover more proteins, which means MNC is able to predict many novel complexes. Moreover, with random initialization, we usually need to repeat the entire calculation multiple times to mitigate the risk of local minimization. Therefore, we suggest devising an effective initialization method rather than initializing *H* randomly.

**Table 1:** Performance of MNC with different initialize method

Methods	# complexes	# proteins	Reference sets			
			CYC2008		SGD	
			Evaluation metrics			
			Acc	FRAC	Acc	FRAC
MNC	1048	3038	0.697	0.726	0.651	0.648
MNC _*rand*_	597	1952	0.695	0.685	0.652	0.609

Here “# complexes”denotes the number of complexes predicted by each algorithm, and “# proteins”denotes the number of proteins covered by the complexes predicted by each algorithm. MNC _*rand*_ corresponds to the results of MNC with random initial conditions

**Fig. 4:**
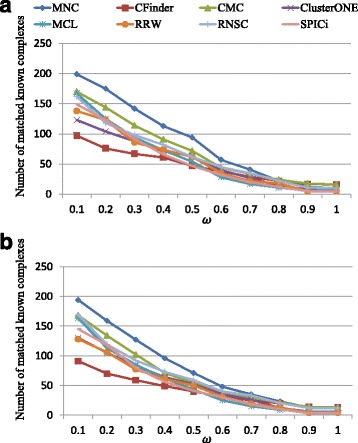
Performance of existing algorithms and MNC in protein complex detection. Amounts of known protein complexes in reference sets (**a**) CYC2008 and (**b**) SGD that are recognized by various algorithms under varying OS threshold *ω*

In addition, for each algorithm, we also calculate the number of known complexes in CYC2008 and SGD reference sets that are recognized by various algorithms under varying OS threshold *ω*, and show the corresponding results in Fig. 4. The number of matched known protein complexes of our MNC algorithm is dramatically higher than that of the other algorithms when *ω* ranges from 0.1 to 0.6. In particular, with respect to SGD reference set, when *ω*=0.2, MNC obtains 159 matched known protein complexes, which is 127%, 18.7%, 51.4%, 40.7%, 33.6%, 50% and 34.7% greater than that achieved by Cfinder, CMC, ClusterONE, MCL, RNSC, RRW and SPICi, respectively. Overall, MNC can predicted more true complexes than other seven classic algorithms.

### Function enrichment analysis

Since the reference complexes sets are incomplete, to further validate the effectiveness of our model, we investigate the biological significance of our predicted protein complexes. Each predicted complex is associated with a *P*-value (as formulated in Eq. (15)) for Gene Ontology (GO) annotation. Note that for each predicted complex, we use the smallest *P*-value over all possible functional groups (i.e., the total GO functions of all the three subontologies, including Biological Process, Cellular Component and Molecular Function are used) to measure its functional homogeneity. The lower the *P*-value is, the stronger biological significance the predicted complex possesses. In this study, we consider a predicted complex to be biologically significant if its *P*-value is less than 1e-2. The web service of GO Term Finder (http://go.princeton.edu/cgi-bin/GOTermFinder) is used to calculate the *P*-value with Bonferroni correction for each predicted complex. The number and percentage of the predicted complexes whose *P*-value falls within [0, 1E-15], [1E-15, 1E-10], [1E-10, 1E-5], [1E-5, 1E-2], [1E-2, 1] are listed in Table 2. We also list the results of CMC since it can achieve the second best performance among all the compared methods. We can find from Table 2 that more than 70% of our predicted complexes are biologically significant, which indicates the effectiveness of our model in detecting functional significant clusters. The results shown in Table 2 also demonstrate that compared to CMC, our MNC can predict more complexes that have *P*-value less than 1E-15, 1E-10, 1E-5 or 1E-2. Table 3 provides 10 protein complexes predicted by MNC that have strong biological significance. The fifth column in Table 3 refers to the number and percentage of proteins in the predicted complex that annotated with the main annotation of GO terms out of the total number of proteins in that complex.

**Table 2:** The number and percentage of the complexes predicted by MNC and CMC that have *P*-value falls within different intervals

Methods	*P*-value				
	< 1E(-15)	1E(-15) to 1E(-10)	1E(-10) to 1E(-5)	1E(-5) to 1E(-2)	1E(-2) to 1
MNC	50 (4.8%)	56 (5.3%)	199 (19%)	476 (45.4%)	267 (25.5%)
CMC	30 (7.3%)	26 (6.3%)	79 (19.2%)	173 (42%)	104 (25.2%)

**Table 3:** Ten predicted protein complexes with smallest *P*-values

Index	*P*-value	Predicted protein complexes	Gene ontology term	Cluster frequency
2	1.21e-31	YCR035C, YDL111C, YDR280W, YGR095C	polyadenylation-dependent	12 out of 14
		YHR069C, YHR081W, YNL189W, YNL232W	snoRNA 3’-end processing	genes, 85.7%
		YOR001W, YOR076C, YGR158C, YGR195W		
		YOL021C, YOL142W		
5	8.98e-31	YAL043C, YDR195W, YDR228C, YDR301W	mRNA polyadenylation	13 out of 17
		YJL033W, YJR093C, YKL018W, YKL059C		genes, 76.5%
		YLR277C, YMR061W, YNL317W, YOR179C		
		YKR002W, YLR115W, YER133W, YGR156W		
		YPR107C		
7	5.85e-32	YBR146W, YBR251W, YDR036C, YDR041W	organellar small ribosomal	14 out of 15
		YGL129C, YGR084C, YHL004W, YIL093C	subunit	genes, 93.3%
		YNL137C, YNL306W, YPL118W, YDR347W		
		YJR113C, YKL155C, YDR337W		
10	3.70-43	YBR217W, YBR272C, YDL007W, YDL097C	proteasome complex	20 out of 21
		YDR427W, YEL037C, YER012W, YER021W		genes, 95.2%
		YFR052W, YGL004C, YGL048C, YHL030W		
		YOR259C, YOR261C, YPR108W, YHR200W		
		YFR004W, YFR010W, YDL147W, YDR394W		
		YKL145W		
13	1.65e-35	YBR119W, YDL087C, YDR235W, YDR240C	U1 snRNP	14 out of 16
		YHR086W, YIL061C, YKL012W, YLR147C		genes, 87.5%
		YML046W, YMR125W, YPL178W, YPR182W		
		YLR275W, YLR298C, YFL017W-A, YGR013W		
18	4.7e-29	YBR254C, YDR108W, YDR246W, YDR407C	TRAPP complex	10 out of 11
		YGR166W, YJL044C, YKR068C, YML077W		genes, 90.9%
		YMR218C, YOR115C, YDR472W		
27	7.34e-36	YBR055C, YBR152W, YDL098C, YDR473C	U4/U6 x U5 tri-snRNP	15 out of 15
		YJR022W, YKL173W, YLR147C, YLR275W	complex	genes, 100%
		YPR082C, YPR178W, YPR182W, YFL017W-A		
		YGR091W, YOR159C, YOR308C		
35	2.05e-30	YBL084C, YDL008W, YDR118W, YFR036W	anaphase-promoting	11 out of 11
		YHR166C, YKL022C, YLR102C, YLR127C	complex	genes, 100%
		YNL172W, YOR249C, YGL240W		
46	9.34e-32	YBL093C, YBR193C, YBR253W, YCR081W	transcription factor activity,	16 out of 17
		YDR443C, YER022W, YGL025C, YGR104C	RNA polymerase II	genes, 94.1%
		YNL236W, YNR010W, YOL051W, YOL135C	transcription factor	
		YHR041C, YHR058C, YDL005C, YDR308C	binding	
		YOR174W		
399	2.77e-28	YBR127C, YDL185W, YEL051W, YGR020C	proton-transporting ATPase	11 out of 11
		YKL080W, YLR447C, YMR054W, YOR270C	activity, rotational mechanism	genes, 100%
		YOR332W, YPR036W, YHR039C-A		

### A case study: the GINS complex

**Fig. 5:**
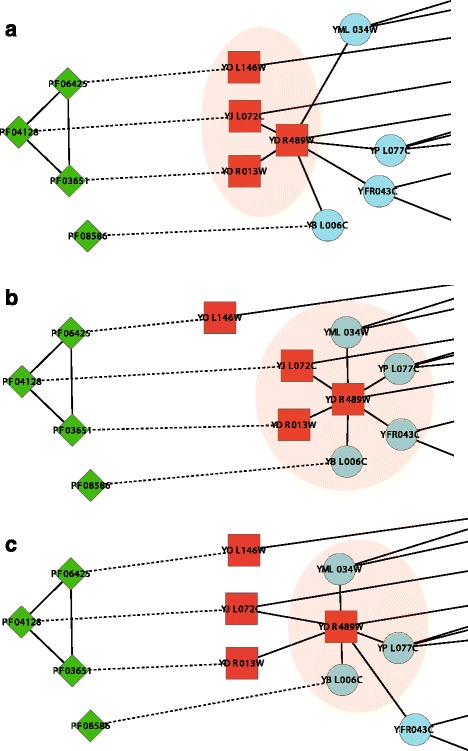
The GINS complex as detected by different computational methods. The shadow area shows the complex predicted by each method (**a**) MNC, (**b**) MCL and (**c**) SPICi. Red rectangle nodes represent subunits of the GINS complex in CYC2008, blue circle nodes represent proteins with other functions and green diamond nodes represent protein domains. The solid lines between nodes represent the interactions between proteins (or protein domains). The dash lines between nodes represent the interactions between proteins and protein domains

In order to illustrate the benefits of integrating multiple heterogeneous networks, we introduce an example of protein complex that can be more accurately identified by MNC. GINS complex in CYC2008 involves 4 proteins, namely, YDR489W, YDR013W, YJL072C and YOL146W. Figure 5 shows how this complex is discovered by the clustering algorithms we have studied. Proteins (or protein domains) that have interactions are connected by solid lines, while the associations between proteins and protein domains are represented by dash lines. Shaded areas represent the clusters detected by various algorithms. Among all the compared algorithms, MNC is the only algorithm that can correctly cover all the proteins in this complex. We can find from Fig. 5 that there are only two interactions among the four protein subunits of GINS complex. Thus, for computational methods that are designed to detect protein complexes from PPI data, it is hard to identify this complex accurately. For instance, MCL can only detect three protein subunits of GINS complex (i.e., YDR489W, YDR013W and YJL072C) and misclassify four proteins into this complex. SPICi is only able to detect one protein subunit of GINS complex, i.e., YDR489W. Since none of the clusters predicted by CFinder, CMC, ClusterONE, RNSC and RRW matched with this complex, their results are not shown here. As shown in Fig. 5, three protein domains, which form a 3-clique in the DDI network, are associated with the protein subunits of GINS complex (i.e., PF06425 is associated with YOL146W, PF04128 is associated with YJL072C and PF03651 is associated with YDR013W). By taking into account domain-protein associations and domain-domain interactions, MNC can accurately identify GINS complex.

## Discussions and conclusions

The joint analysis of multiple heterogeneous network data has the potential to increase the accuracy of protein complex detection. In this study, a novel multi-network clustering (MNC) model is developed to integrate multiple heterogeneous networks for protein complex detection. Our MNC model could make use of the cross-field relationships between proteins and protein domains to guide the search of protein complexes. Experiment comparisons on two real-world data sets show that our MNC outperforms other state-of-the-art protein complex detection methods in terms of two evaluation metrics with respect to three benchmark complex sets. These results show the effect of domain-domain interactions on protein complex identification, which suggests that the domain information should be used if it is available. Our model is a flexible framework, which can also be used to solve some multi-view learning problems. Regarding the future works, we would like to design an algorithm which can incorporate more types of data, including homogeneous and heterogeneous network data for protein complex detection.
